# Interleukin (IL)-17A Inhibitor as a Treatment for Darier Disease in the Pediatric Population

**DOI:** 10.7759/cureus.100826

**Published:** 2026-01-05

**Authors:** Bianka D Barajas Mendez, Guadalupe Maldonado-Colin, Lucia Achell Nava, Mariela R Rosas Garcia, Alexis Rivas Antonio

**Affiliations:** 1 Department of Dermatology, Universidad Nacional Autonoma de Mexico, Mexico City, MEX; 2 Department of Dermatology, Centro Medico Nacional 20 de Noviembre, Instituto de Seguridad y Servicios Sociales de los Trabajadores del Estado, Mexico City, MEX; 3 Department of Internal Medicine, Universidad del Ejercito y Fuerza Aérea, Mexico City, MEX

**Keywords:** anti-il17 therapy, darier disease, genodermatosis, pediatric dermatology, refractory disease

## Abstract

Darier disease is a genodermatosis that significantly affects patients’ quality of life. We present the case of a 16-year-old patient whose quality of life remained markedly impaired despite systemic treatment. The patient experienced substantial clinical improvement in cutaneous lesions and quality of life following treatment with an interleukin (IL)-17A inhibitor. IL-17A inhibitors have demonstrated benefit in adults with Darier disease. In this case, we also observed therapeutic efficacy in a pediatric patient, supporting the relevance of this treatment approach in younger individuals. IL-17A inhibitors may play an important role in the management of Darier disease and represent a promising therapeutic option for pediatric patients, particularly in cases refractory to conventional therapies.

## Introduction

Darier disease is a genodermatosis caused by mutations in the ATPase sarcoplasmic/endoplasmic reticulum Ca2+ transporting 2 (ATP2A2) gene, which encodes sarco(endo)plasmic reticulum calcium-ATPase 2 (SERCA2), which is responsible for calcium transport at the cellular level [1]. It has a reported global prevalence of 1:30,000 to 1:100,000. It is an autosomal dominant disorder with complete penetrance and variable expression, with spontaneous mutation in 68% of patients [2]. Recent studies have postulated the importance of the inflammatory response with an increase in the response of B lymphocytes directly affected by the ATP2A2 gene [3] with a significant increase in T helper (TH)17 lymphocytes and interleukin (IL)-17, IL-23 [4].

This condition is characterized by the formation of hyperkeratotic, malodorous, oily, yellow or brown plaques that are mainly located in seborrheic areas [5]. The main symptoms are pruritus, burning, and pain [2]. The diagnosis is made based on clinical suspicion and subsequent biopsy, where it is common to find hyperkeratosis and hypergranulosis, with suprabasal acantholysis and epidermal papillary hyperplasia, and dyskeratotic cells in the stratum corneum and granular layer, described as round bodies [1,5].

There is no consensus on treatment. Effective topical treatments such as retinoids and 5-fluorouracil have been reported, as well as systemic treatments for patients with extensive disease using retinoids, cyclosporine, and doxycycline [6]. In recent years, the importance of the TH17 response in inflammatory skin diseases has been demonstrated, with IL-17 and IL-23 playing a major role, leading to new therapies targeting these cytokines [4]. Treatment with IL-17A inhibitors has shown significant improvement in lesions in patients who have failed systemic therapies [4].

## Case presentation

**Figure 1:**
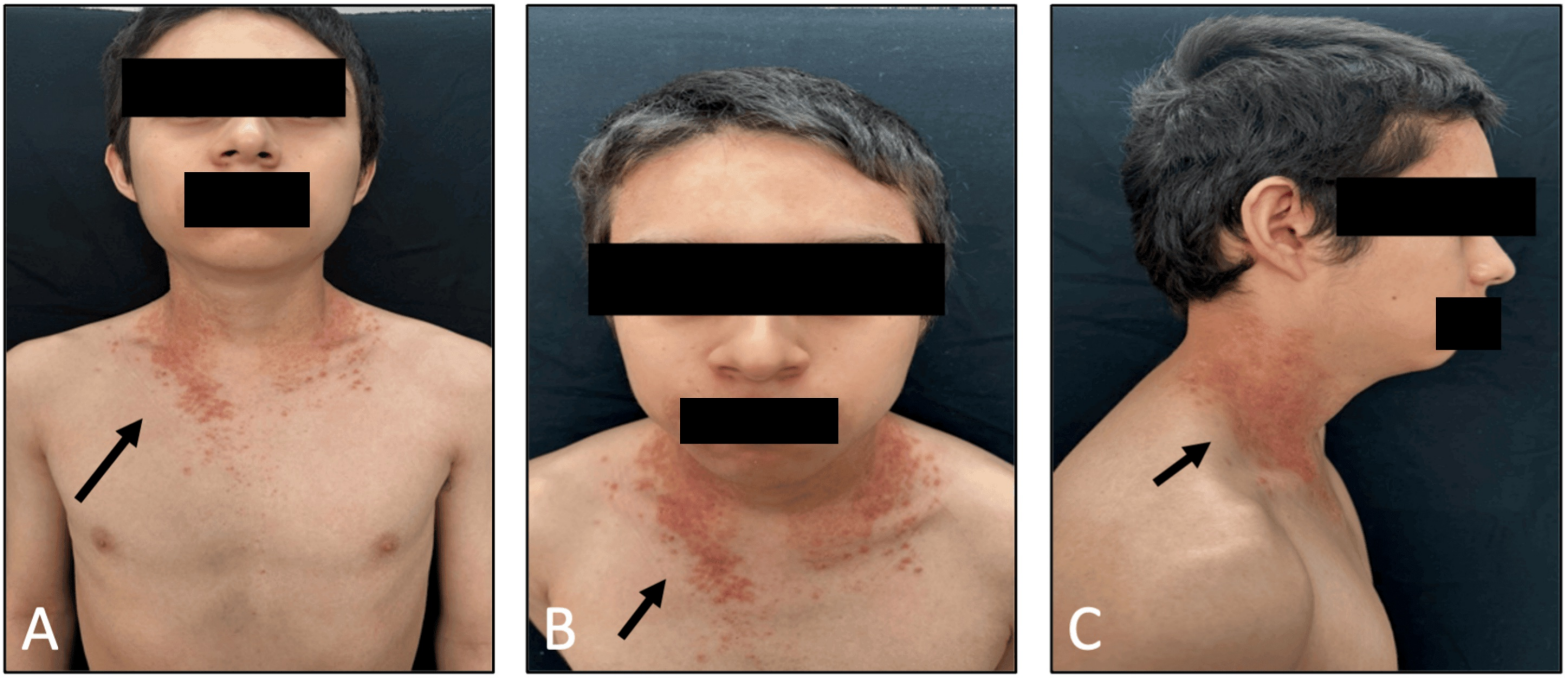
Manifestations of Darier disease in the adolescent patient (A) Hyperkeratotic, oily plaques (B and C) Dermatosis localized on the neck and hairline, multiple hyperkeratotic papules.

**Figure 2:**
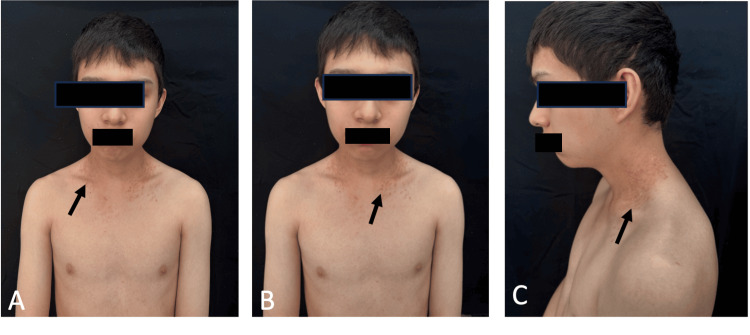
The patient's images after treatment with interleukin-17A inhibitor A. Darier disease five weeks after starting treatment with an interleukin-17A inhibitor. B. Reduction in hyperkeratotic papules in the neck area. C. Lateral neck region with improvement in dermatosis.

This is a case of a 16-year-old adolescent with dermatosis since the age of nine, located in the hairline, neck, and pelvis regions, consisting of irregularly shaped red scaly erythematous plaques with a rough surface and defined edges (Figures 1A-1C). In the comprehensive evaluation of the patient, he was classified with a Dermatology Life Quality Index (DLQI) [7], which is assessed on a scale of 0-15; a skin condition with a score of 0-1 has no impact on the patient's life, 2-5 minor impact, 6-10 moderate impact, 11-20 severe impact and 21-30 very severe impact on the patient's life. In this case, the patient's DLQI was 14, with a severe impact on quality of life, and an Investigator's Global Assessment (IGA) [8] (0 clear, 1 almost clear, 2 mild, 3 moderate, and 4 severe). The patient's IGA score was 3, which classifies it as a moderate disease. A biopsy was performed, which reported intraepidermal vesicular dermatitis due to acantholysis and dyskeratosis compatible with Darier's disease. Treatment was initiated with oral isotretinoin at 40 mg once daily. A monthly follow-up was performed, with no improvement in the dermatosis observed. Therefore, it was decided to add an IL-17A inhibitor to her treatment with secukinumab 300 mg weekly for five weeks, at an induction dose, and subsequently secukinumab 300 mg every four weeks as a maintenance dose. The patient was monitored during the induction dose, and improvement was observed in the fifth week of treatment, when he presented with a dermatosis consisting of irregularly shaped, pink, scaly erythematous plaques with diffuse edges. He had a DLQI score of 3 with a small impact on quality of life and an IGA score of 1, classifying him as having an almost clear disease (Figures 2A-2C).

## Discussion

Darier disease is a genodermatosis that affects patients from an early age, with a worldwide prevalence of 1:30,000 to 1:100,000. Patients present clinical alterations with a significant impact on quality of life. It is chronic and difficult to treat in most cases, which poses a medical challenge. It's a chronic and often treatment-resistant condition, representing a significant medical challenge [1,4]. In a study conducted by Kositkuljorn and Suchonwanit, the clinical presentation of Darier disease was characterized by multiple skin-colored, hyperkeratotic papules that coalesced to form plaques, resulting in marked epidermal thickening with prominent ridges and furrows. However, compared to the classic topography presented in this clinical class, there is evidence of facial locations and regions exposed to the sun [9].

Recent studies have demonstrated the importance of immunological alterations in patients with Darier disease, allowing them to be classified according to their molecular phenotype, mainly directed at the TH17 response [4]. In one of the studies, Giorgio et al. reported a series of cases of patients diagnosed with Darier disease treated with IL-17A inhibitor (brodalumab), in which three of the four patients reported significant improvement six months after starting treatment, and the fourth patient reported moderate improvement, secondary to the severity of the clinical manifestations [10]. The clinical case presented in this article is consistent with the clinical response observed in patients treated with brodalumab. Both studies were conducted with IL-17A inhibitors, which confirms the importance of this interleukin in Darier disease. It is important to mention that, in this study, the patients were adults, the youngest being 26 years old at the time of the study, since brodalumab is currently only approved for use in individuals over 18 years [10]. This limits therapeutic options for pediatric patients to secukinumab and ixekinumab, which are currently approved for this group of patients with conditions such as psoriasis [11].

Ettinger et al. (2023) reported a study in which patients with Darier disease were classified according to the profile of affected cytokines, the main ones being IL-17 and IL-23. Treatment was adjusted based on these parameters, and treatment was initiated with an adequate response to these two biological therapies [4], which is consistent with the results obtained in the present study, in which the patient showed significant clinical improvement with an interleukin IL-17A inhibitor.

There are other types of treatments, such as apremilast, which blocks the activity of TH1 and TH17 lymphocytes, leading to a decrease in interleukins such as CXCL10, interferon-γ, IL-23, and IL-17. Muto et al. conducted a study with this treatment in which, as in our case report, inhibiting these signaling pathways led to a significant improvement in Darier's disease lesions. However, as in the previous case reports, these studies were conducted in a population over 18 years of age, which differs from the present study, which was conducted in a pediatric population [12]. Our study presents a novel therapeutic option, as there are currently insufficient case reports in the pediatric population diagnosed with Darier disease.

## Conclusions

IL-17A inhibition may offer an important therapeutic option for pediatric patients with Darier disease who do not experience adequate control with conventional treatments. In this case, the introduction of an IL-17A inhibitor resulted in notable clinical improvement, including a reduction in lesion burden and improved quality of life, suggesting a meaningful benefit in disease management. From a clinical standpoint, this option may be considered in patients who continue to present active disease despite appropriate systemic therapy. The patient showed improvement in dermatosis by the third week of induction doses, with a response assessed by DLQI from 14 to 3 and IGA from 3 to 1. It is therefore expected that in the future, this will be considered part of the treatment for this group of patients.
